# To the Dental Profession

**Published:** 1876-03

**Authors:** 


*To the Dental Profession.*—The American Academy of Dental Science, Boston, Mass., design presenting to the profession and the public, at the approaching Centennial, a view of American Dentistry,—past and present. One of the characteristics of this presentation will be a history of the profession in this country, particularly for the past one hundred years. This work being designed to become a standard of reference, will be comprehensive and complete in detail.

To the end that this desirable result may be attained, the committee in charge respectfully request the aid of all dentists, (and others,) in furnishing information of every kind necessary to the work.

Below will be found a list designed to afford a general idea of the kinds of information needed, and on these and kindred subjects, and indeed on any subject connected with the profession—nothing will be regarded as too small or insignificant to be of value.

If desired, any material received, will be carefully preserved, and returned to the sender after use. To facilitate this, as well as ensure credit being properly given, everything should be marked with the name and address of the sender.

*List of Subjects.*—Biography. Literature. Societies. Colleges. Individuals. Mechanical and Operative Dentistry. Discoveries in any branch of the profession. Materia Medica. Prominent events (with dates.) Prominent Questions. Inventions. Patents. Anecdotes. Sayings. Historical Facts. Obituaries. Addresses. Papers. Published and unpublished works, etc., etc.

Address communications and material, to Geo. T. Moffatt, M. D., No. 1, Hotel Boylston, Boston, *or to* James E. Dexter, care W. A. Bronson, M. D., No. 8 East 34th Street, New York city.

